# Supplementary material for: Genetic Dissection of Phosphorus Use Efficiency in a Maize Association Population under Two P Levels in the Field
Source: Int J Mol Sci. 2021 Aug 27;22(17):9311. doi: 10.3390/ijms22179311 (PMC8430673; doi:10.3390/ijms22179311)
Supplement: Supplementary file 1 [file ijms-22-09311-s001.zip › Supplementary files_proofreading_20210827/Caption of supplementary files.pdf]

Figures:

Figure S1. Distribution and interaction of traits under low-P and normal-P trials. **(A)** YPP. **(B)** SePCc. **(C)** SDWPP. **(D)** ShPCc. **(E)** ADWPP. YPP: Yield per plant; SePCc: Seed P concentration; SDWPP: Shoot dry weight per plant; ShPCc: Shoot P concentration; ADWPP: All dry weight per plant. The significant difference was calculated by t-tests between the phenotypes under low-P and normal-P conditions for all traits.

Figure S2. The correlations of LPTI for eleven traits. YPP: Yield per plant; SDWPP: Shoot dry weight per plant; ADWPP: All dry weight per plant; SePCc: Seed P concentration; ShPCc: Shoot P concentration; SePCPP: Seed P content per plant; ShPCPP: Shoot P content per plant; APCPP: P content per plant; SePUtE: Seed P utilization efficiency; ShPUtE: Shoot P utilization efficiency; APUtE: All P utilization efficiency. *: $p < 0.05$; **: $p < 0.01$; ***: $p < 0.001$.

Figure S3. The boxplots of four subgroups of five traits under low-P condition. **(A)** YPP. **(B)** SePCc. **(C)** SDWPP. **(D)** ShPCc. **(E)** ADWPP. These four subpopulations are mixed (n = 101), non-stiff stalk (NSS, n = 118), stiff stalk (SS, n = 29), tropical/subtropical (TST, n = 111). Multiple comparisons were conducted by the LSD test method at a 0.05 significance level. Different letters represent significant differences. The rhombus in the boxplot is the mean value. YPP: Yield per plant; SePCc: Seed P concentration; SDWPP: Shoot dry weight per plant; ShPCc: Shoot P concentration; ADWPP: All dry weight per plant.

Figure S4. Manhattan (left) and quantile-quantile (right) plot of eleven traits under low-P condition. **(A)** YPP. **(B)** SDWPP. **(C)** ADWPP. **(D)** SePCc. **(E)** ShPCc. **(F)** SePCPP. **(G)** ShPCPP. **(H)** APCPP. **(I)** SePUtE. **(J)** APUtE. YPP: Yield per plant; SDWPP: Shoot dry weight per plant; ADWPP: All dry weight per plant; SePCc: Seed P concentration; ShPCc: Shoot P concentration; SePCPP: Seed P content per plant; ShPCPP: Shoot P content per plant; APCPP: P content per plant; SePUtE: Seed P utilization efficiency; ShPUtE: Shoot P utilization efficiency; APUtE: All P utilization efficiency.

Figure S5. Manhattan (left) and quantile-quantile (right) plot of eleven traits under normal-P condition. **(A)** YPP. **(B)** SDWPP. **(C)** ADWPP. **(D)** SePCc. **(E)** ShPCc. **(F)** SePCPP. **(G)** ShPCPP. **(H)** APCPP. **(I)** SePUtE. **(J)** ShPUtE. **(K)** APUtE. YPP: Yield per plant; SDWPP: Shoot dry weight per plant; ADWPP: All dry weight per plant; SePCc: Seed P concentration; ShPCc: Shoot P concentration; SePCPP: Seed P content per plant; ShPCPP: Shoot P content per plant; APCPP: P content per plant; SePUtE: Seed P utilization efficiency; ShPUtE: Shoot P utilization efficiency; APUtE: All P utilization efficiency.

Figure S6. Manhattan (left) and quantile-quantile (right) plot for LPTI of eleven traits across both P levels. **(A)** YPP. **(B)** SDWPP. **(C)** ADWPP. **(D)** SePCc. **(E)** ShPCc. **(F)** SePCPP. **(G)** ShPCPP. **(H)** APCPP. **(I)** SePUtE. **(J)** ShPUtE. **(K)** APUtE. YPP: Yield per plant; SDWPP: Shoot dry weight per plant; ADWPP: All dry weight per plant; SePCc: Seed P concentration; ShPCc: Shoot P concentration; SePCPP: Seed P content per plant; ShPCPP: Shoot P content per plant; APCPP: P content per plant; SePUtE: Seed P utilization efficiency; ShPUtE: Shoot P utilization efficiency; APUtE: All P utilization efficiency.

Figure S7. The structure of GRMZM5G848945 and pairwise LD analysis. The dotted line is the significant threshold of 4.94.

Figure S8. The structure of GRMZM2G030762 and pairwise LD analysis. The dotted line is the significant threshold of 4.94.

Figure S9. The prediction ability of GBLUP of eleven traits in the low-P, normal-P and LPTI datasets. Yield per plant; SDWPP: Shoot dry weight per plant; ADWPP: All dry weight per plant; SePCc: Seed P concentration; ShPCc: Shoot P concentration; SePCPP: Seed P content per plant; ShPCPP: Shoot P content per plant; APCPP: P content per plant; SePUtE: Seed P utilization efficiency; ShPUtE: Shoot P utilization efficiency; APUtE: All P utilization efficiency.

Tables:

Table S1. Candidate genes of 11 traits under the two P conditions and LPTI dataset

Table S2. Information of PHT1 gene family in four species

Table S3. A description of traits in this study
