# Supplementary material for: Genetic Dissection of Phosphorus Use Efficiency in a Maize Association Population under Two P Levels in the Field
Source: Int J Mol Sci. 2021 Aug 27;22(17):9311. doi: 10.3390/ijms22179311 (PMC8430673; doi:10.3390/ijms22179311)
Supplement: Supplementary file 1 [file ijms-22-09311-s001.zip › Supplementary files_proofreading_20210827/Table S1.pdf]

Table S1. Summary significant SNP of all traits under the two P conditions and LPTI

SNP	Chr.	position	$-\log_{10} P$	Trait	Condition	Gene	Maker position
chr2.S_215941022	2	216609992	5.16	APCPP	LP	GRMZM2G158766	WithinGene
chr8.S_119905885	8	119432925	5.1	APCPP	LP	GRMZM2G004703	WithinGene
chr8.S_119905885	8	119432925	5.1	APCPP	LP	GRMZM5G895362	WithinGene
chr8.S_153045260	8	152583441	5.51	APCPP	LP	GRMZM2G032484	WithinGene
chr8.S_153045337	8	152583518	5.51	APCPP	LP	GRMZM2G032484	WithinGene
chr8.S_153045506	8	152583687	5.51	APCPP	LP	GRMZM2G032484	WithinGene
chr8.S_153047685	8	152585866	5.51	APCPP	LP	GRMZM2G032484	WithinGene
chr8.S_153047929	8	152586110	5.36	APCPP	LP	GRMZM2G032484	WithinGene
chr2.S_197529123	2	198182721	5.48	APUtE	LP	GRMZM2G312501	WithinGene
chr7.S_146244617	7	146280765	5.09	APUtE	LP	GRMZM2G042666	WithinGene
chr7.S_146244618	7	146280766	5.09	APUtE	LP	GRMZM2G042666	WithinGene
chr3.S_140852927	3	140881141	5.05	ADWPP	LP	GRMZM2G463464	WithinGene
chr3.S_140853493	3	140881707	5.05	ADWPP	LP	GRMZM2G463464	WithinGene
chr3.S_140857207	3	140885421	5.16	ADWPP	LP	GRMZM2G463464	WithinGene
chr2.S_196383637	2	197033528	5.44	SePCc	LP	GRMZM2G151122	WithinGene
chr5.S_205781313	5	205838208	5.13	SePCc	LP	GRMZM2G140150	WithinGene
chr8.S_168286541	8	167837049	5.25	SePCc	LP	GRMZM2G149230	WithinGene
chr8.S_168287312	8	167837820	5.27	SePCc	LP	GRMZM2G149230	WithinGene
chr8.S_168287510	8	167838018	5.25	SePCc	LP	GRMZM2G149230	WithinGene
chr8.S_168287559	8	167838067	5.25	SePCc	LP	GRMZM2G149230	WithinGene
chr8.S_168289605	8	167840113	5.25	SePCc	LP	GRMZM2G149230	WithinGene
chr8.S_168290170	8	167840678	5.25	SePCc	LP	GRMZM2G149230	WithinGene
chr8.S_168290213	8	167840721	5.44	SePCc	LP	GRMZM2G149230	WithinGene
chr8.S_168290214	8	167840722	5.44	SePCc	LP	GRMZM2G149230	WithinGene
chr8.S_168290216	8	167840724	5.44	SePCc	LP	GRMZM2G149230	WithinGene
chr8.S_168290339	8	167840847	5.25	SePCc	LP	GRMZM2G149230	WithinGene
chr1.S_28643451	1	28644019	5.12	SePCPP	LP	GRMZM2G040828	WithinGene
chr3.S_5580300	3	5581618	7.03	SePCPP	LP	GRMZM2G143235	WithinGene
chr5.S_21933463	5	21954744	5.43	SePCPP	LP	GRMZM2G096372	WithinGene
chr8.S_162559636	8	162109084	7.17	SePCPP	LP	GRMZM2G030762	WithinGene
chr9.S_26819179	9	26832567	7.87	SePCPP	LP	GRMZM2G475293	WithinGene
chr10.S_26090270	10	26098212	7.06	SePCPP	LP	GRMZM2G050394	WithinGene
chr10.S_77270047	10	77283397	5.4	SePCPP	LP	GRMZM2G110145	WithinGene
chr2.S_196383637	2	197033528	5.11	SePUtE	LP	GRMZM2G151122	WithinGene
chr5.S_205781313	5	205838208	4.95	SePUtE	LP	GRMZM2G140150	WithinGene
chr1.S_5532257	1	5525913	5	ShPCc	LP	GRMZM2G055704	WithinGene
chr1.S_5532257	1	5525913	5	ShPCc	LP	GRMZM5G802002	WithinGene
chr2.S_44201877	2	44830522	11.13	ShPCc	LP	GRMZM2G171254	WithinGene
chr2.S_44201877	2	44830522	11.13	ShPCc	LP	GRMZM2G171277	WithinGene
chr2.S_216686343	2	217355313	5.67	ShPCc	LP	GRMZM2G001297	WithinGene
chr9.S_4221767	9	4221990	5.16	ShPCc	LP	GRMZM2G563453	WithinGene
chr5.S_61803436	5	61831685	4.98	ShPCPP	LP	GRMZM2G069923	WithinGene

chr5.S_175732995	5	175779671	5.59	ShPCPP	LP	GRMZM2G025236	WithinGene
chr6.S_128467569	6	128650528	5.03	ShPCPP	LP	GRMZM2G019180	WithinGene
chr6.S_128467669	6	128650628	5.03	ShPCPP	LP	GRMZM2G019180	WithinGene
chr6.S_128467673	6	128650632	5.03	ShPCPP	LP	GRMZM2G019180	WithinGene
chr6.S_128467713	6	128650672	4.95	ShPCPP	LP	GRMZM2G019180	WithinGene
chr6.S_128467740	6	128650699	4.95	ShPCPP	LP	GRMZM2G019180	WithinGene
chr6.S_128483844	6	128666803	5.41	ShPCPP	LP	GRMZM2G008252	WithinGene
chr6.S_128483844	6	128666803	5.41	ShPCPP	LP	AC207798.2_FG003	WithinGene
chr6.S_128483856	6	128666815	4.97	ShPCPP	LP	GRMZM2G008252	WithinGene
chr6.S_128483856	6	128666815	4.97	ShPCPP	LP	AC207798.2_FG003	WithinGene
chr8.S_153045260	8	152583441	5.68	ShPCPP	LP	GRMZM2G032484	WithinGene
chr8.S_153045337	8	152583518	5.68	ShPCPP	LP	GRMZM2G032484	WithinGene
chr8.S_153045506	8	152583687	5.68	ShPCPP	LP	GRMZM2G032484	WithinGene
chr8.S_153047685	8	152585866	5.49	ShPCPP	LP	GRMZM2G032484	WithinGene
chr8.S_153047929	8	152586110	5.18	ShPCPP	LP	GRMZM2G032484	WithinGene
chr2.S_44040366	2	44669011	5.63	ShPUtE	LP	GRMZM2G703021	WithinGene
chr2.S_44201861	2	44830506	5.04	ShPUtE	LP	GRMZM2G171254	WithinGene
chr2.S_44201861	2	44830506	5.04	ShPUtE	LP	GRMZM2G171277	WithinGene
chr2.S_44201877	2	44830522	5.49	ShPUtE	LP	GRMZM2G171254	WithinGene
chr2.S_44201877	2	44830522	5.49	ShPUtE	LP	GRMZM2G171277	WithinGene
chr2.S_44201924	2	44830569	5.7	ShPUtE	LP	GRMZM2G171254	WithinGene
chr2.S_44201924	2	44830569	5.7	ShPUtE	LP	GRMZM2G171277	WithinGene
chr2.S_44553517	2	45182162	5.17	ShPUtE	LP	GRMZM2G046402	WithinGene
chr2.S_44553561	2	45182206	5.17	ShPUtE	LP	GRMZM2G046402	WithinGene
chr2.S_44553619	2	45182264	5.17	ShPUtE	LP	GRMZM2G046402	WithinGene
chr2.S_44553622	2	45182267	5.17	ShPUtE	LP	GRMZM2G046402	WithinGene
chr2.S_44554556	2	45183201	5.17	ShPUtE	LP	GRMZM2G046402	WithinGene
chr2.S_44554774	2	45183419	5.17	ShPUtE	LP	GRMZM2G046402	WithinGene
chr2.S_44557153	2	45185798	5.17	ShPUtE	LP	GRMZM2G046402	WithinGene
chr2.S_44557480	2	45186125	5.17	ShPUtE	LP	GRMZM2G046402	WithinGene
chr2.S_44561004	2	45189649	5.17	ShPUtE	LP	GRMZM2G046402	WithinGene
chr2.S_44561123	2	45189768	5.17	ShPUtE	LP	GRMZM2G046402	WithinGene
chr2.S_44561635	2	45190280	5.17	ShPUtE	LP	GRMZM2G046402	WithinGene
chr2.S_44561636	2	45190281	5.17	ShPUtE	LP	GRMZM2G046402	WithinGene
chr2.S_44561637	2	45190282	5.17	ShPUtE	LP	GRMZM2G046402	WithinGene
chr2.S_44561639	2	45190284	5.17	ShPUtE	LP	GRMZM2G046402	WithinGene
chr2.S_45523411	2	46152056	4.97	ShPUtE	LP	GRMZM2G131321	WithinGene
chr2.S_45523465	2	46152110	5.23	ShPUtE	LP	GRMZM2G131321	WithinGene
chr2.S_45523880	2	46152525	4.98	ShPUtE	LP	GRMZM2G131321	WithinGene
chr2.S_45523898	2	46152543	5.23	ShPUtE	LP	GRMZM2G131321	WithinGene
chr2.S_45524407	2	46153052	4.97	ShPUtE	LP	GRMZM2G131448	WithinGene
chr2.S_45524576	2	46153221	5.23	ShPUtE	LP	GRMZM2G131448	WithinGene
chr2.S_45525833	2	46154478	4.97	ShPUtE	LP	GRMZM2G131448	WithinGene
chr2.S_65038237	2	65668640	5.15	ShPUtE	LP	GRMZM5G848945	WithinGene

chr2.S_65038546	2	65668949	5.07	ShPUtE	LP	GRMZM5G848945	WithinGene
chr2.S_65038633	2	65669036	5.07	ShPUtE	LP	GRMZM5G848945	WithinGene
chr2.S_65038693	2	65669096	5.07	ShPUtE	LP	GRMZM5G848945	WithinGene
chr5.S_31881708	5	31901549	5	ShPUtE	LP	GRMZM2G326707	WithinGene
chr5.S_32921625	5	32941366	5.26	ShPUtE	LP	GRMZM2G465728	WithinGene
chr5.S_32921626	5	32941367	5.26	ShPUtE	LP	GRMZM2G465728	WithinGene
PZE-101210110	1	259440409	5.13	SDWPP	LP	GRMZM2G038677	WithinGene
chr2.S_233744569	2	234414624	5.58	SDWPP	LP	GRMZM5G878490	WithinGene
chr3.S_140847548	3	140875762	7.54	SDWPP	LP	GRMZM2G163476	InterGene
chr3.S_140847548	3	140875762	7.54	SDWPP	LP	GRMZM2G463464	InterGene
chr3.S_189468910	3	189527658	12.58	SDWPP	LP	GRMZM5G801031	WithinGene
chr3.S_219398419	3	219482603	4.94	SDWPP	LP	GRMZM2G061723	WithinGene
chr4.S_198119781	4	198297622	4.95	SDWPP	LP	GRMZM2G067555	WithinGene
chr4.S_198119790	4	198297631	4.95	SDWPP	LP	GRMZM2G067555	WithinGene
chr4.S_234211637	4	234750870	5.01	SDWPP	LP	GRMZM2G084296	WithinGene
chr7.S_136178412	7	136212881	5.1	SDWPP	LP	GRMZM5G867125	WithinGene
chr8.S_104461014	8	103983158	9.75	SDWPP	LP	GRMZM2G047152	WithinGene
chr9.S_89707199	9	90713476	6.58	SDWPP	LP	GRMZM2G442551	WithinGene
chr9.S_89707222	9	90713499	6.58	SDWPP	LP	GRMZM2G442551	WithinGene
PZE-109051911	9	90731240	5.35	SDWPP	LP	GRMZM2G567962	WithinGene
chr1.S_43757872	1	43762394	5.96	YPP	LP	GRMZM2G141273	WithinGene
chr2.S_36445844	2	37074813	6.88	YPP	LP	GRMZM2G130109	WithinGene
PZE-109002002	9	2486194	5.29	YPP	LP	GRMZM2G016275	WithinGene
chr10.S_26090270	10	26098212	9.86	YPP	LP	GRMZM2G050394	WithinGene
chr3.S_194053777	3	194117335	11.28	APCPP	NP	GRMZM2G152461	WithinGene
chr3.S_197281874	3	197351697	11.81	APCPP	NP	GRMZM2G069694	WithinGene
chr4.S_90032984	4	90058996	7.31	APCPP	NP	GRMZM2G133969	WithinGene
chr5.S_168223085	5	168263560	7.76	APCPP	NP	GRMZM2G059851	WithinGene
chr7.S_5095645	7	5100589	8.3	APCPP	NP	GRMZM2G011858	WithinGene
chr7.S_104756249	7	104784426	9.95	APCPP	NP	GRMZM2G010389	WithinGene
chr10.S_3275321	10	3278276	7.56	APCPP	NP	GRMZM5G873586	WithinGene
chr1.S_41957970	1	41962492	5	APUtE	NP	GRMZM2G044237	WithinGene
chr1.S_207321298	1	207365547	5.19	APUtE	NP	GRMZM2G460383	WithinGene
chr2.S_44201861	2	44830506	10.04	APUtE	NP	GRMZM2G171254	WithinGene
chr2.S_44201861	2	44830506	10.04	APUtE	NP	GRMZM2G171277	WithinGene
PZE-102108812	2	140278799	5.72	APUtE	NP	GRMZM2G066213	InterGene
chr4.S_186482132	4	186657630	8.31	APUtE	NP	GRMZM5G877788	WithinGene
chr5.S_1528326	5	1528609	9.5	APUtE	NP	GRMZM2G448001	WithinGene
chr8.S_6027557	8	6027997	5.56	APUtE	NP	GRMZM2G049416	WithinGene
chr1.S_43236278	1	43240800	5.16	ADWPP	NP	GRMZM2G109967	WithinGene
chr2.S_205904801	2	206568370	5.31	ADWPP	NP	GRMZM2G099622	WithinGene
chr2.S_205905553	2	206569122	5.42	ADWPP	NP	GRMZM2G099622	WithinGene
chr2.S_205922590	2	206586159	5.45	ADWPP	NP	GRMZM2G099463	WithinGene
chr3.S_168921028	3	168964362	5.45	ADWPP	NP	GRMZM5G807064	WithinGene

chr3.S_168921028	3	168964362	5.45	ADWPP	NP	GRMZM5G806744	WithinGene
chr4.S_90018830	4	90044842	5.19	ADWPP	NP	GRMZM2G133969	WithinGene
chr4.S_92059573	4	92085485	5	ADWPP	NP	GRMZM2G068239	WithinGene
chr4.S_191046114	4	191224055	11.25	ADWPP	NP	GRMZM2G123355	WithinGene
chr5.S_169842157	5	169885156	5.52	ADWPP	NP	GRMZM5G864335	WithinGene
chr7.S_103877574	7	103905751	5.07	ADWPP	NP	GRMZM2G315431	WithinGene
chr8.S_149888168	8	149423590	5.04	ADWPP	NP	GRMZM2G060516	WithinGene
chr8.S_149888168	8	149423590	5.04	ADWPP	NP	AC196465.3_FG002	WithinGene
chr1.S_81639965	1	81649853	5.2	SePCc	NP	GRMZM2G066369	WithinGene
chr1.S_290306123	1	290378327	5.38	SePCc	NP	GRMZM2G082312	WithinGene
chr2.S_40504144	2	41132889	4.97	SePCc	NP	GRMZM2G042443	WithinGene
chr3.S_170545810	3	170589144	5.09	SePCc	NP	GRMZM2G011731	WithinGene
chr4.S_69341509	4	69366402	5.22	SePCc	NP	AC205521.3_FG003	WithinGene
chr4.S_69342136	4	69367029	5.22	SePCc	NP	AC205521.3_FG003	WithinGene
chr6.S_153744795	6	153938652	4.99	SePCc	NP	GRMZM2G100639	WithinGene
PUT-163a-76012177-3730	1	295507524	5.3	SePCPP	NP	GRMZM2G156756	WithinGene
PUT-163a-76012177-3731	1	295507707	5.28	SePCPP	NP	GRMZM2G156756	WithinGene
chr3.S_157018077	3	157060281	8.5	SePCPP	NP	GRMZM5G898668	WithinGene
chr4.S_224957242	4	225495586	4.95	SePCPP	NP	GRMZM2G048733	WithinGene
chr5.S_7775885	5	7783875	9.77	SePCPP	NP	GRMZM2G077295	WithinGene
chr5.S_173128200	5	173172610	7.11	SePCPP	NP	GRMZM2G418916	WithinGene
chr8.S_172464084	8	172017061	12.17	SePCPP	NP	GRMZM2G111511	WithinGene
chr9.S_132885020	9	133138263	5.61	SePCPP	NP	GRMZM2G102382	WithinGene
chr10.S_9532676	10	9537083	8.15	SePCPP	NP	GRMZM2G324131	InterGene
chr1.S_290306123	1	290378327	5.29	SePUtE	NP	GRMZM2G082312	WithinGene
chr4.S_69341509	4	69366402	5.53	SePUtE	NP	AC205521.3_FG003	WithinGene
chr4.S_69342136	4	69367029	5.53	SePUtE	NP	AC205521.3_FG003	WithinGene
chr9.S_151260007	9	151526421	5.48	SePUtE	NP	GRMZM2G178741	WithinGene
chr2.S_44201861	2	44830506	11.96	ShPCc	NP	GRMZM2G171254	WithinGene
chr2.S_44201861	2	44830506	11.96	ShPCc	NP	GRMZM2G171277	WithinGene
chr5.S_70045964	5	70074665	5.2	ShPCc	NP	GRMZM2G098420	WithinGene
chr5.S_213819919	5	213868600	11.54	ShPCc	NP	GRMZM2G100467	WithinGene
chr6.S_156930328	6	157124806	7.73	ShPCc	NP	GRMZM2G015100	WithinGene
chr7.S_7280158	7	7285102	5.14	ShPCc	NP	GRMZM2G107309	WithinGene
SYN23809	8	164952669	8.97	ShPCc	NP	GRMZM2G119314	WithinGene
chr1.S_262321452	1	262381182	5.21	ShPCPP	NP	GRMZM2G080725	WithinGene
chr9.S_148202483	9	148460442	5.52	ShPCPP	NP	GRMZM2G181540	WithinGene
chr9.S_148202494	9	148460453	5.24	ShPCPP	NP	GRMZM2G181540	WithinGene
chr2.S_44201861	2	44830506	7.34	ShPUtE	NP	GRMZM2G171254	WithinGene
chr2.S_44201861	2	44830506	7.34	ShPUtE	NP	GRMZM2G171277	WithinGene
SYN1397	5	189452222	7.09	ShPUtE	NP	GRMZM2G315121	WithinGene
chr5.S_213819919	5	213868600	5.04	ShPUtE	NP	GRMZM2G100467	WithinGene
SYN23809	8	164952669	5.59	ShPUtE	NP	GRMZM2G119314	WithinGene
chr8.S_165402790	8	164952690	4.96	ShPUtE	NP	GRMZM2G119314	WithinGene

chr8.S_165402818	8	164952718	5.21	ShPUtE	NP	GRMZM2G119314	WithinGene
chr8.S_165403065	8	164952965	5.21	ShPUtE	NP	GRMZM2G119314	WithinGene
chr8.S_165403068	8	164952968	5.21	ShPUtE	NP	GRMZM2G119314	WithinGene
chr8.S_165403374	8	164953274	5.16	ShPUtE	NP	GRMZM2G119314	WithinGene
chr8.S_165403485	8	164953385	5.29	ShPUtE	NP	GRMZM2G119314	WithinGene
chr8.S_165403659	8	164953559	5.46	ShPUtE	NP	GRMZM2G119314	WithinGene
chr8.S_165404620	8	164954520	5.46	ShPUtE	NP	GRMZM2G119314	WithinGene
chr8.S_165404683	8	164954583	5.46	ShPUtE	NP	GRMZM2G119314	WithinGene
chr8.S_165404728	8	164954628	5.46	ShPUtE	NP	GRMZM2G119314	WithinGene
chr8.S_165404768	8	164954668	5.16	ShPUtE	NP	GRMZM2G119314	WithinGene
chr4.S_191045769	4	191223710	5.29	SDWPP	NP	GRMZM2G123355	WithinGene
chr4.S_191046111	4	191224052	5.63	SDWPP	NP	GRMZM2G123355	WithinGene
chr4.S_191046113	4	191224054	5.63	SDWPP	NP	GRMZM2G123355	WithinGene
chr4.S_191046114	4	191224055	5.7	SDWPP	NP	GRMZM2G123355	WithinGene
chr4.S_191046115	4	191224056	5.7	SDWPP	NP	GRMZM2G123355	WithinGene
chr4.S_191046116	4	191224057	5.7	SDWPP	NP	GRMZM2G123355	WithinGene
chr5.S_18066742	5	18088123	5.79	SDWPP	NP	GRMZM2G146599	WithinGene
chr5.S_152743178	5	152782199	6.42	SDWPP	NP	GRMZM2G453575	WithinGene
chr2.S_44201877	2	44830522	5.34	YPP	NP	GRMZM2G171254	WithinGene
chr2.S_44201877	2	44830522	5.34	YPP	NP	GRMZM2G171277	WithinGene
chr4.S_228033906	4	228572345	5.01	YPP	NP	GRMZM2G345055	WithinGene
chr5.S_12272921	5	12284418	4.97	YPP	NP	GRMZM2G102754	WithinGene
SYN11171	1	80081626	5.06	APCPP	LPTI	GRMZM2G064804	WithinGene
chr1.S_161775544	1	161791514	4.95	APCPP	LPTI	GRMZM2G703781	InterGene
chr1.S_161775544	1	161791514	4.95	APCPP	LPTI	AF546187.1_FG001	InterGene
chr1.S_161775544	1	161791514	4.95	APCPP	LPTI	AF546187.1_FG002	InterGene
chr1.S_161775555	1	161791525	4.95	APCPP	LPTI	GRMZM2G703781	InterGene
chr1.S_161775555	1	161791525	4.95	APCPP	LPTI	AF546187.1_FG001	InterGene
chr1.S_161775555	1	161791525	4.95	APCPP	LPTI	AF546187.1_FG002	InterGene
chr1.S_161776194	1	161792164	5.42	APCPP	LPTI	GRMZM2G703781	InterGene
chr1.S_161776194	1	161792164	5.42	APCPP	LPTI	AF546187.1_FG001	InterGene
chr1.S_161776194	1	161792164	5.42	APCPP	LPTI	AF546187.1_FG002	InterGene
chr1.S_161776212	1	161792182	4.95	APCPP	LPTI	GRMZM2G703781	InterGene
chr1.S_161776212	1	161792182	4.95	APCPP	LPTI	AF546187.1_FG001	InterGene
chr1.S_161776212	1	161792182	4.95	APCPP	LPTI	AF546187.1_FG002	InterGene
chr2.S_207840133	2	208503702	5.77	APCPP	LPTI	GRMZM2G431309	WithinGene
chr2.S_207840139	2	208503708	5.77	APCPP	LPTI	GRMZM2G431309	WithinGene
chr4.S_224234273	4	224772617	5.14	APCPP	LPTI	GRMZM2G016923	WithinGene
PZE-104137492	4	224774870	5.14	APCPP	LPTI	GRMZM2G016923	WithinGene
chr7.S_7954766	7	7959710	5.68	APCPP	LPTI	GRMZM2G021149	WithinGene
chr7.S_7958420	7	7963364	5.68	APCPP	LPTI	GRMZM2G178496	WithinGene
chr7.S_7958427	7	7963371	5.68	APCPP	LPTI	GRMZM2G178496	WithinGene
chr8.S_175368631	8	174922558	5.12	APCPP	LPTI	GRMZM2G567165	WithinGene
chr1.S_39747873	1	39752395	5.01	APUtE	LPTI	GRMZM5G828820	WithinGene

chr1.S_39747874	1	39752396	5.01	APUtE	LPTI	GRMZM5G828820	WithinGene
chr3.S_10674683	3	10680854	5.15	APUtE	LPTI	GRMZM2G074580	WithinGene
chr8.S_90791443	8	90301570	6.45	APUtE	LPTI	GRMZM2G093186	WithinGene
chr1.S_273899522	1	273966524	5.58	ADWPP	LPTI	GRMZM2G361593	WithinGene
chr1.S_273899522	1	273966524	5.58	ADWPP	LPTI	GRMZM5G863590	WithinGene
chr1.S_273899582	1	273966584	5.58	ADWPP	LPTI	GRMZM2G361593	WithinGene
chr1.S_273899582	1	273966584	5.58	ADWPP	LPTI	GRMZM5G863590	WithinGene
chr2.S_176452629	2	177098542	5.27	ADWPP	LPTI	GRMZM2G158502	WithinGene
chr2.S_176452693	2	177098606	5.57	ADWPP	LPTI	GRMZM2G158502	WithinGene
chr2.S_176452809	2	177098722	5.42	ADWPP	LPTI	GRMZM2G158502	WithinGene
chr3.S_132396238	3	132423414	5.27	ADWPP	LPTI	GRMZM2G063949	WithinGene
chr4.S_224909946	4	225448290	5.06	ADWPP	LPTI	GRMZM2G174598	WithinGene
chr1.S_37133693	1	37138215	5.67	SePCc	LPTI	GRMZM5G883632	WithinGene
chr1.S_37133694	1	37138216	5.67	SePCc	LPTI	GRMZM5G883632	WithinGene
chr1.S_37133695	1	37138217	5.67	SePCc	LPTI	GRMZM5G883632	WithinGene
chr1.S_37133696	1	37138218	5.67	SePCc	LPTI	GRMZM5G883632	WithinGene
chr1.S_37133697	1	37138219	5.41	SePCc	LPTI	GRMZM5G883632	WithinGene
chr2.S_222588610	2	223257909	6.31	SePCc	LPTI	GRMZM2G155849	WithinGene
chr2.S_222589848	2	223259147	4.96	SePCc	LPTI	GRMZM2G155849	WithinGene
chr2.S_14715146	2	14726491	5.24	SePCPP	LPTI	GRMZM2G421256	WithinGene
chr4.S_6073942	4	6078678	5.29	SePCPP	LPTI	GRMZM5G832890	WithinGene
chr6.S_168920663	6	169128066	5.71	SePCPP	LPTI	GRMZM2G127426	WithinGene
chr8.S_29982167	8	29986970	5.28	SePCPP	LPTI	GRMZM2G072791	WithinGene
chr4.S_234211683	4	234750916	4.99	SePUtE	LPTI	GRMZM2G533536	InterGene
chr4.S_234211683	4	234750916	4.99	SePUtE	LPTI	GRMZM2G084296	InterGene
chr4.S_234211683	4	234750916	4.99	SePUtE	LPTI	GRMZM2G084314	InterGene
chr4.S_234406903	4	234946136	5.16	SePUtE	LPTI	GRMZM2G039848	WithinGene
chr6.S_2720972	6	2723860	5.21	SePUtE	LPTI	GRMZM2G523025	InterGene
chr6.S_2720972	6	2723860	5.21	SePUtE	LPTI	GRMZM2G068854	InterGene
chr6.S_2720972	6	2723860	5.21	SePUtE	LPTI	GRMZM2G367941	InterGene
chr6.S_160234907	6	160430429	4.97	SePUtE	LPTI	GRMZM2G132212	WithinGene
chr7.S_8178542	7	8183486	5.47	ShPCc	LPTI	GRMZM2G076630	WithinGene
chr7.S_8178542	7	8183486	5.47	ShPCc	LPTI	GRMZM2G377761	WithinGene
chr7.S_8178577	7	8183521	5.09	ShPCc	LPTI	GRMZM2G076630	WithinGene
chr7.S_8178577	7	8183521	5.09	ShPCc	LPTI	GRMZM2G377761	WithinGene
chr7.S_8178590	7	8183534	6.51	ShPCc	LPTI	GRMZM2G076630	WithinGene
chr7.S_8178590	7	8183534	6.51	ShPCc	LPTI	GRMZM2G377761	WithinGene
chr8.S_164619610	8	164169058	5.07	ShPCc	LPTI	GRMZM2G080816	WithinGene
chr10.S_3164087	10	3167042	5.67	ShPCc	LPTI	GRMZM2G356817	WithinGene
chr10.S_3164087	10	3167042	5.67	ShPCc	LPTI	GRMZM5G821769	WithinGene
chr10.S_3164219	10	3167174	5.38	ShPCc	LPTI	GRMZM2G356817	WithinGene
chr10.S_3164219	10	3167174	5.38	ShPCc	LPTI	GRMZM5G821769	WithinGene
chr10.S_3164433	10	3167388	4.99	ShPCc	LPTI	GRMZM2G356817	WithinGene
chr10.S_3164433	10	3167388	4.99	ShPCc	LPTI	GRMZM5G821769	WithinGene

chr1.S_68438365	1	68446088	4.95	ShPCPP	LPTI	GRMZM2G003530	WithinGene
chr2.S_63789107	2	64419510	9.39	ShPCPP	LPTI	GRMZM2G001084	WithinGene
chr5.S_23184282	5	23204307	7.79	ShPCPP	LPTI	GRMZM2G339562	WithinGene
SYN38866	5	212368556	4.99	ShPCPP	LPTI	GRMZM5G885644	WithinGene
chr1.S_46865308	1	46869830	5.33	ShPUtE	LPTI	GRMZM2G129302	WithinGene
chr1.S_270558127	1	270624812	5.06	ShPUtE	LPTI	GRMZM2G133183	WithinGene
chr1.S_270558159	1	270624844	5.06	ShPUtE	LPTI	GRMZM2G133183	WithinGene
chr1.S_270558168	1	270624853	5.06	ShPUtE	LPTI	GRMZM2G133183	WithinGene
chr1.S_270558191	1	270624876	5.06	ShPUtE	LPTI	GRMZM2G133183	WithinGene
chr1.S_270558206	1	270624891	5.06	ShPUtE	LPTI	GRMZM2G133183	WithinGene
chr1.S_270558316	1	270625001	5.08	ShPUtE	LPTI	GRMZM2G133183	WithinGene
PZE-102018940	2	8997706	5.01	ShPUtE	LPTI	GRMZM2G000052	WithinGene
chr7.S_8178590	7	8183534	5.15	ShPUtE	LPTI	GRMZM2G076630	WithinGene
chr7.S_8178590	7	8183534	5.15	ShPUtE	LPTI	GRMZM2G377761	WithinGene
chr1.S_105151135	1	105159610	5.1	SDWPP	LPTI	GRMZM5G868757	InterGene
chr1.S_105325156	1	105335709	5.08	SDWPP	LPTI	GRMZM2G457147	WithinGene
chr1.S_273899522	1	273966524	5.62	SDWPP	LPTI	GRMZM2G361593	WithinGene
chr1.S_273899522	1	273966524	5.62	SDWPP	LPTI	GRMZM5G863590	WithinGene
chr1.S_273899582	1	273966584	5.62	SDWPP	LPTI	GRMZM2G361593	WithinGene
chr1.S_273899582	1	273966584	5.62	SDWPP	LPTI	GRMZM5G863590	WithinGene
chr6.S_106351747	6	106520193	5.11	SDWPP	LPTI	GRMZM2G129375	WithinGene
chr6.S_153257357	6	153451214	5.02	SDWPP	LPTI	GRMZM2G169943	WithinGene
chr1.S_290933926	1	291006770	5.56	YPP	LPTI	GRMZM2G104125	WithinGene

Note: Position: The physical position (B73 RefGen_v3) of significant SNP; YPP: Yield per plant; ADWPP: All dry weight per plant; SDWPP: Shoot dry weight per plant; SePCc: Seed P concentration; SePCPP: Seed P content per plant; SePUtE: Seed P utilization efficiency; ShPCc: Shoot P concentration; ShPCPP: Shoot P content per plant; ShPUtE: Shoot P utilization efficiency; APCPP: P content per plant; APUtE: All P utilization efficiency. Maker position: The significant SNPs were in the target gene (WithinGene) or between two genes (InterGene).
