# Supplementary material for: Genetic Dissection of Phosphorus Use Efficiency in a Maize Association Population under Two P Levels in the Field
Source: Int J Mol Sci. 2021 Aug 27;22(17):9311. doi: 10.3390/ijms22179311 (PMC8430673; doi:10.3390/ijms22179311)
Supplement: Supplementary file 1 [file ijms-22-09311-s001.zip › Supplementary files_proofreading_20210827/Table S3.pdf]

Table S3. A description of traits in this study

Trait	Abbreviation	Description
Yield per plant (g)	YPP	Grain yield divide by plant number harvested
Shoot dry weight per plant (g)	SDWPP	Shoot was dried at 65°C to a constant weight and divide by plant number harvested
All dry weight per plant (g)	ADWPP	Dried weight (ear and shoot) divide by plant number harvested
Seed P concentration (mg/g)	SePCc	The P content per gram of dried grain
Shoot P concentration (mg/g)	ShPCc	The P content per gram of dried shoot
Seed P content per plant (mg)	SePCPP	YPP × SePCc
Shoot P content per plant (mg)	ShPCPP	SDWPP × ShPCc
All P content per plant (mg)	APCPP	SePCPP + ShPCPP
Seed P utilization efficiency (g/mg)	SePUtE	inverse of SePCc
Shoot P utilization efficiency (g/mg)	ShPUtE	inverse of ShPCc
All P utilization efficiency (g/mg)	APUtE	ADWPP ÷ APCPP
