# Supplementary figures and images for: Genetic Dissection of Phosphorus Use Efficiency in a Maize Association Population under Two P Levels in the Field
Source: Int J Mol Sci. 2021 Aug 27;22(17):9311. doi: 10.3390/ijms22179311 (PMC8430673; doi:10.3390/ijms22179311)

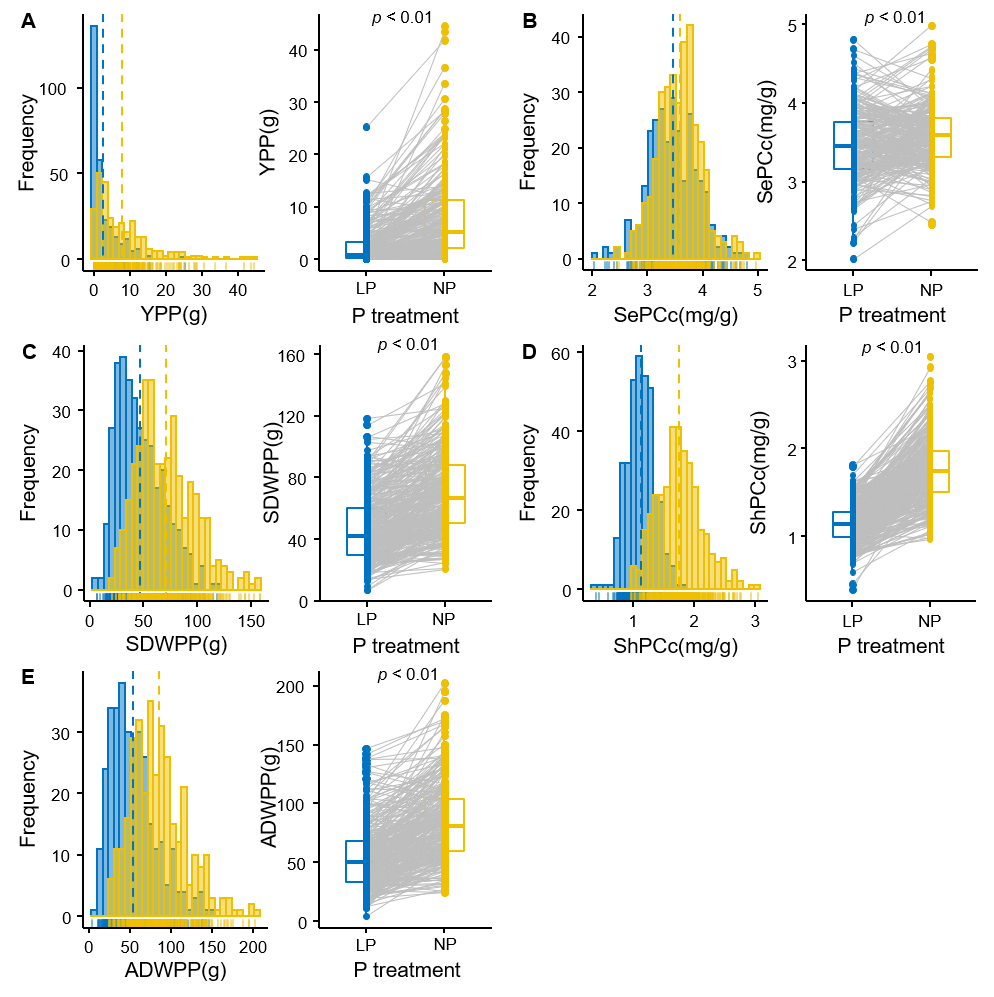

Supplement: Supplementary file 1 [file ijms-22-09311-s001.zip › Supplementary files_proofreading_20210827/Figure S1.png]

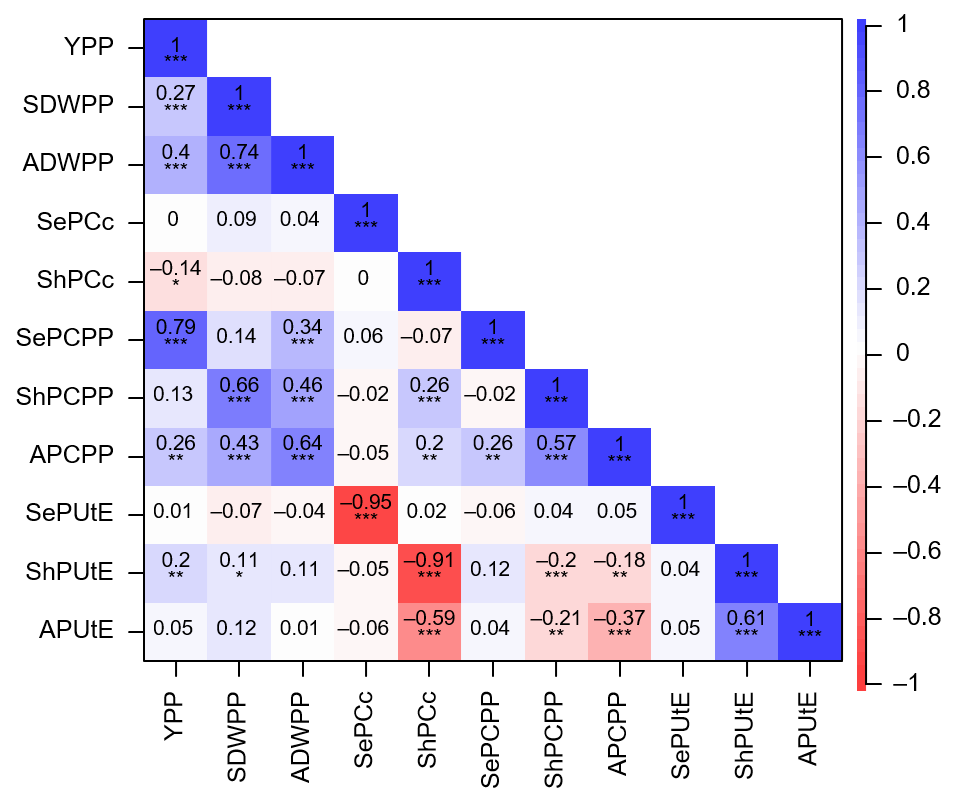

Supplement: Supplementary file 1 [file ijms-22-09311-s001.zip › Supplementary files_proofreading_20210827/Figure S2.png]

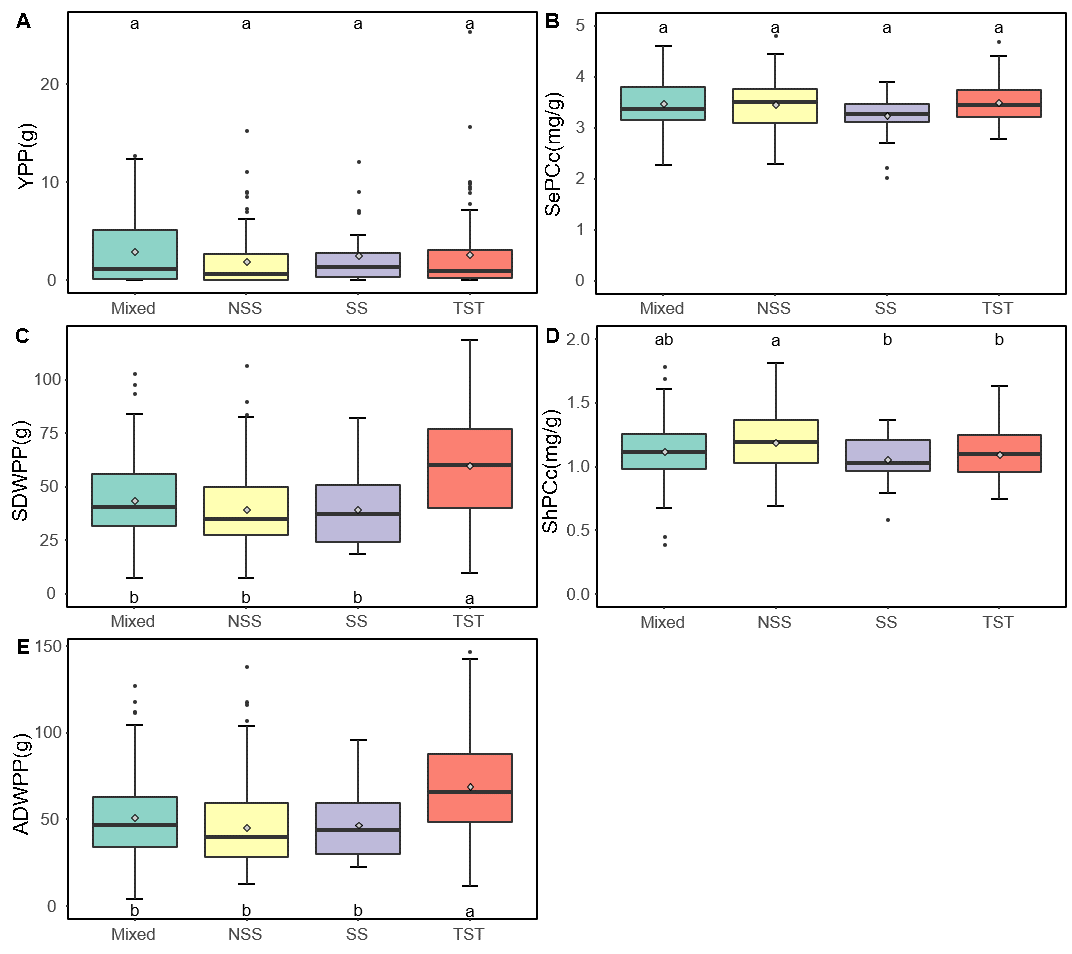

Supplement: Supplementary file 1 [file ijms-22-09311-s001.zip › Supplementary files_proofreading_20210827/Figure S3.png]

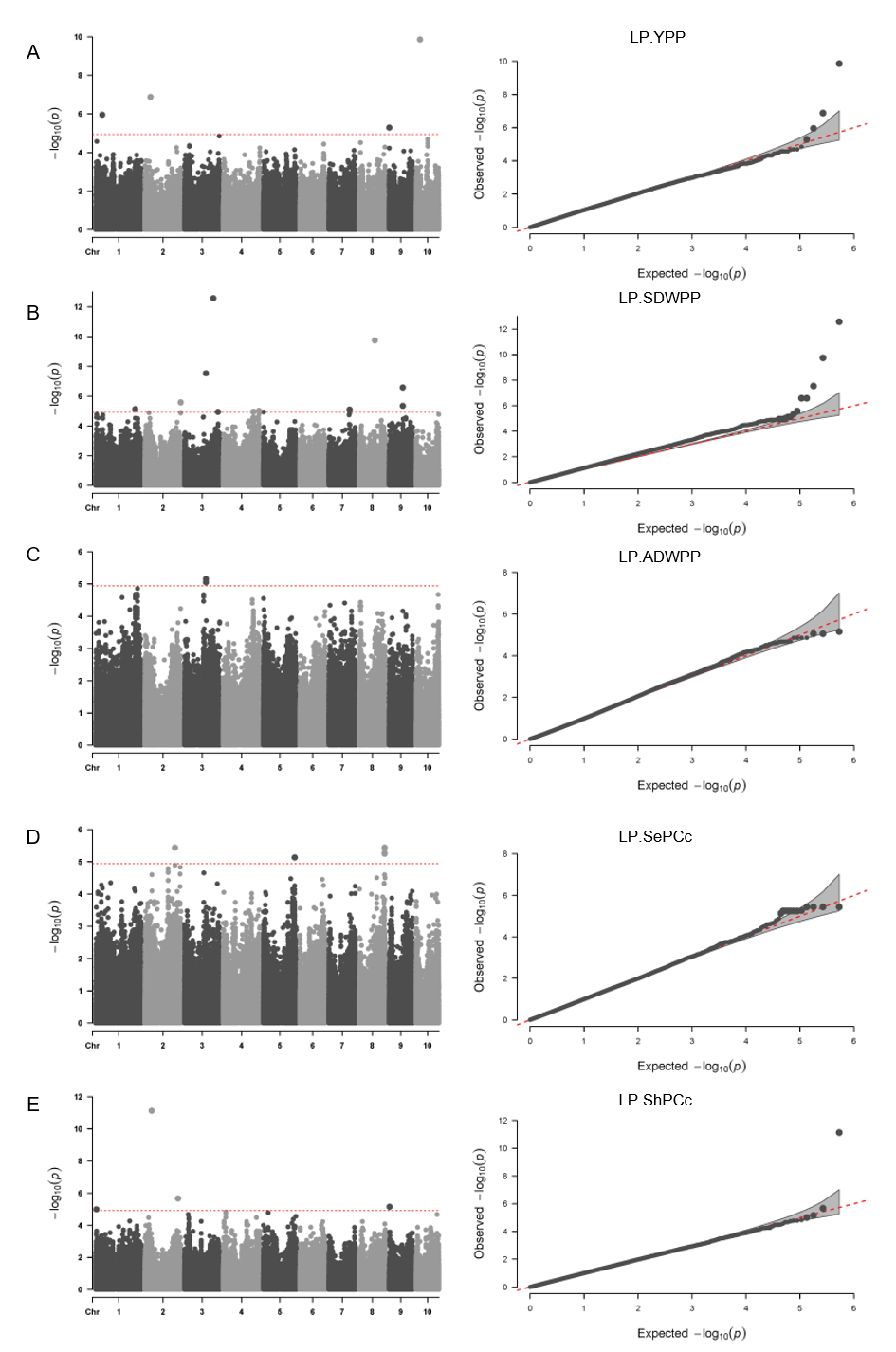

Supplement: Supplementary file 1 [file ijms-22-09311-s001.zip › Supplementary files_proofreading_20210827/Figure S4-Part1.png]

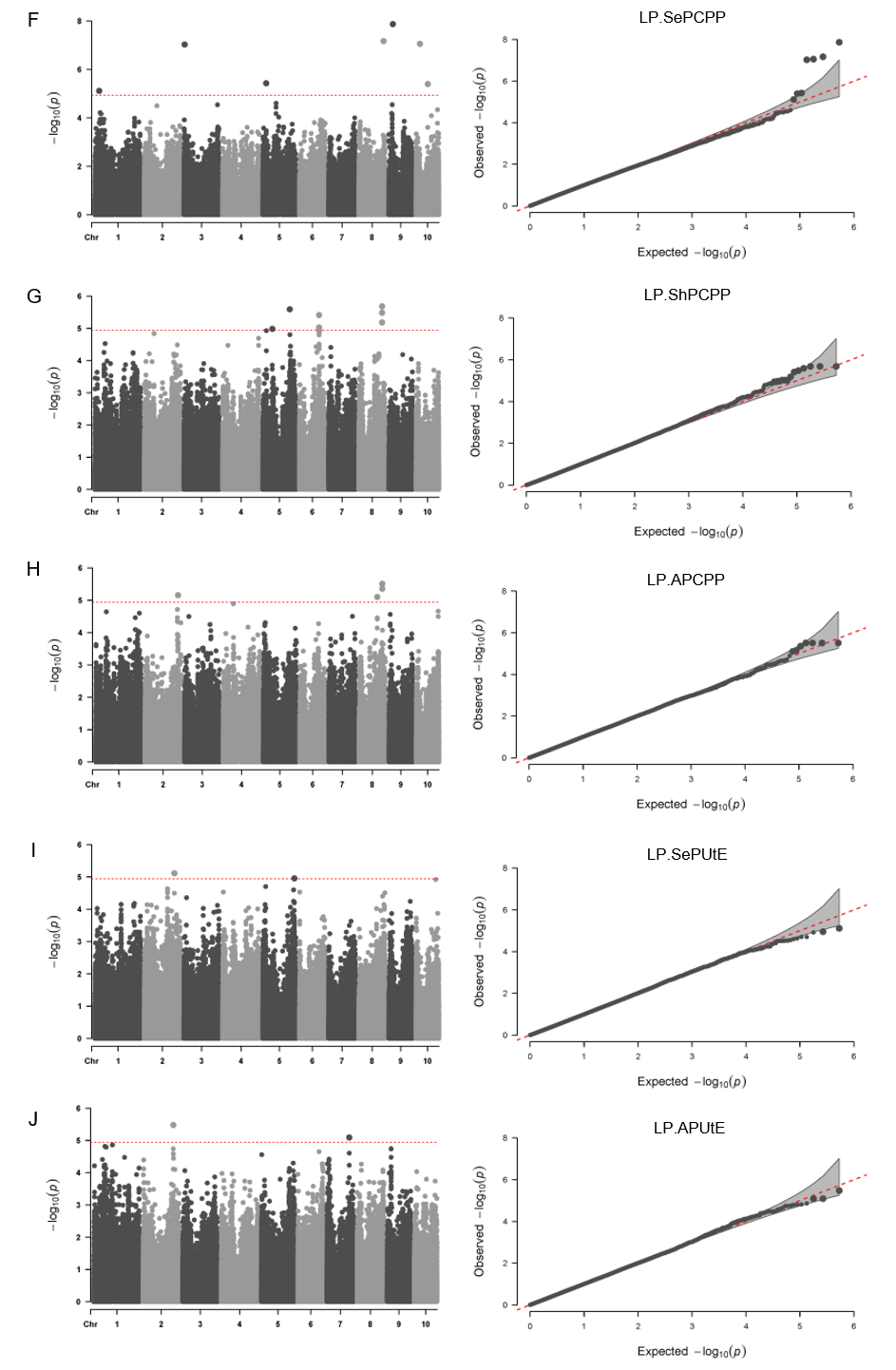

Supplement: Supplementary file 1 [file ijms-22-09311-s001.zip › Supplementary files_proofreading_20210827/Figure S4-Part2.png]

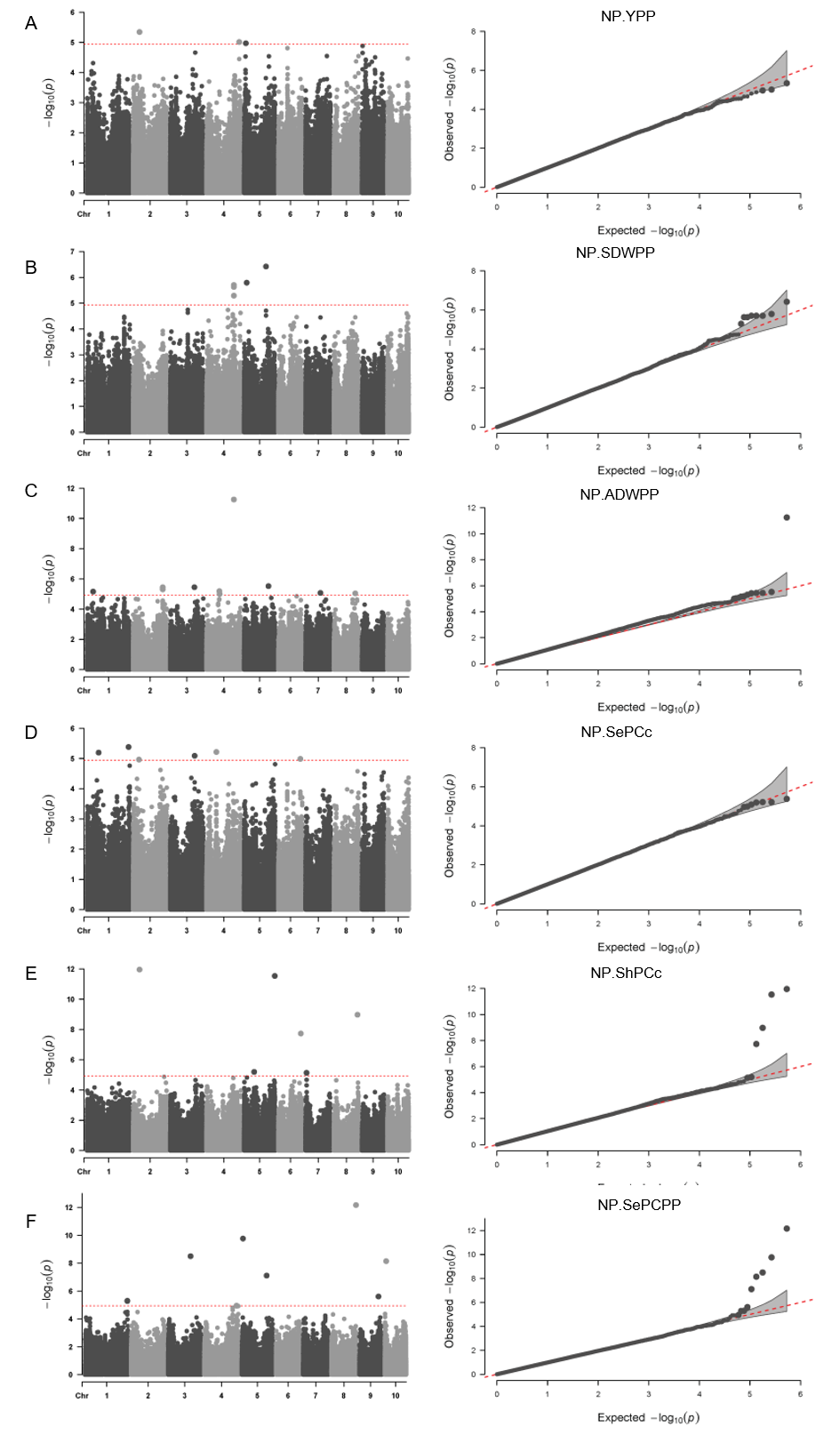

Supplement: Supplementary file 1 [file ijms-22-09311-s001.zip › Supplementary files_proofreading_20210827/Figure S5-Part1.png]

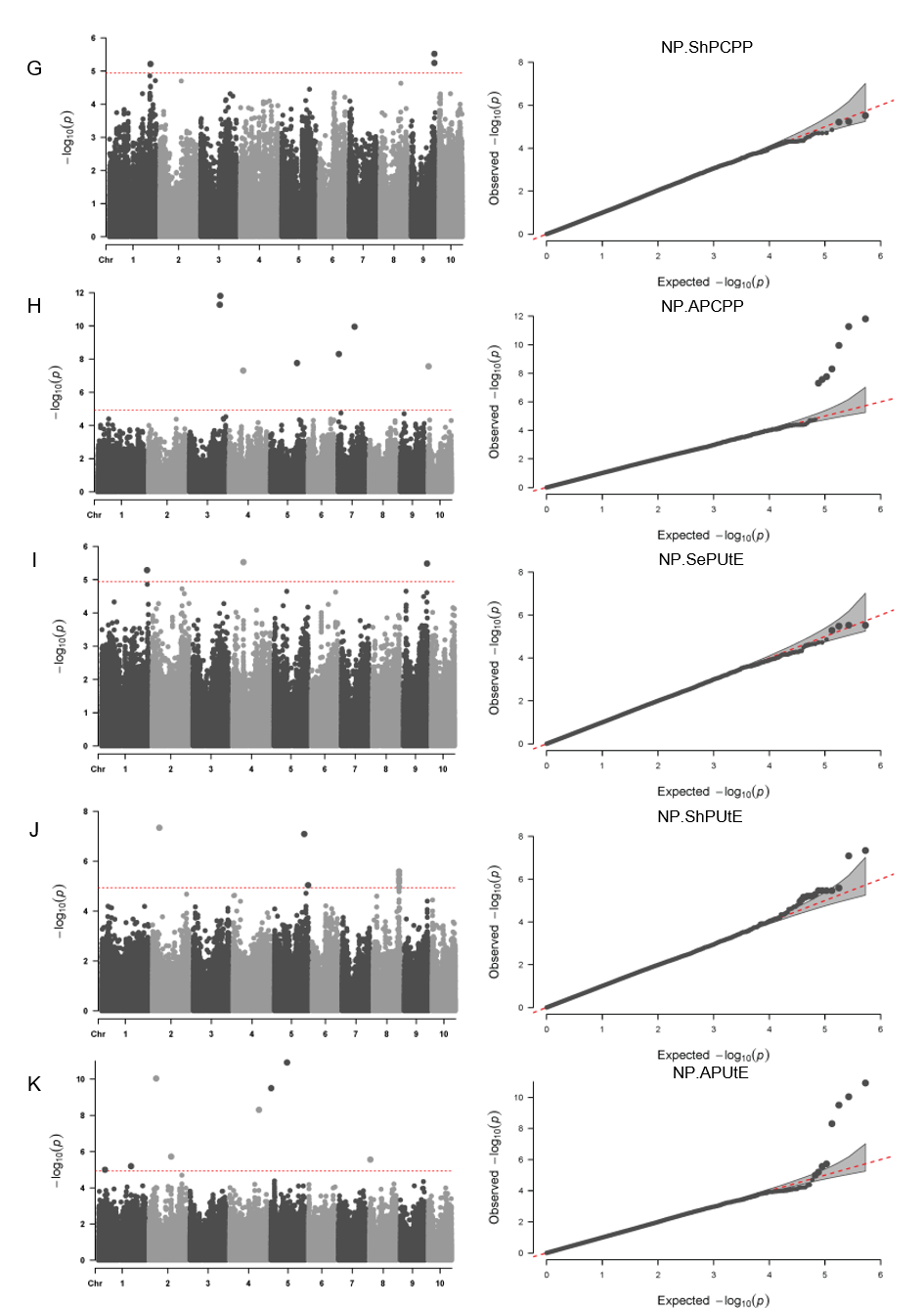

Supplement: Supplementary file 1 [file ijms-22-09311-s001.zip › Supplementary files_proofreading_20210827/Figure S5-Part2.png]

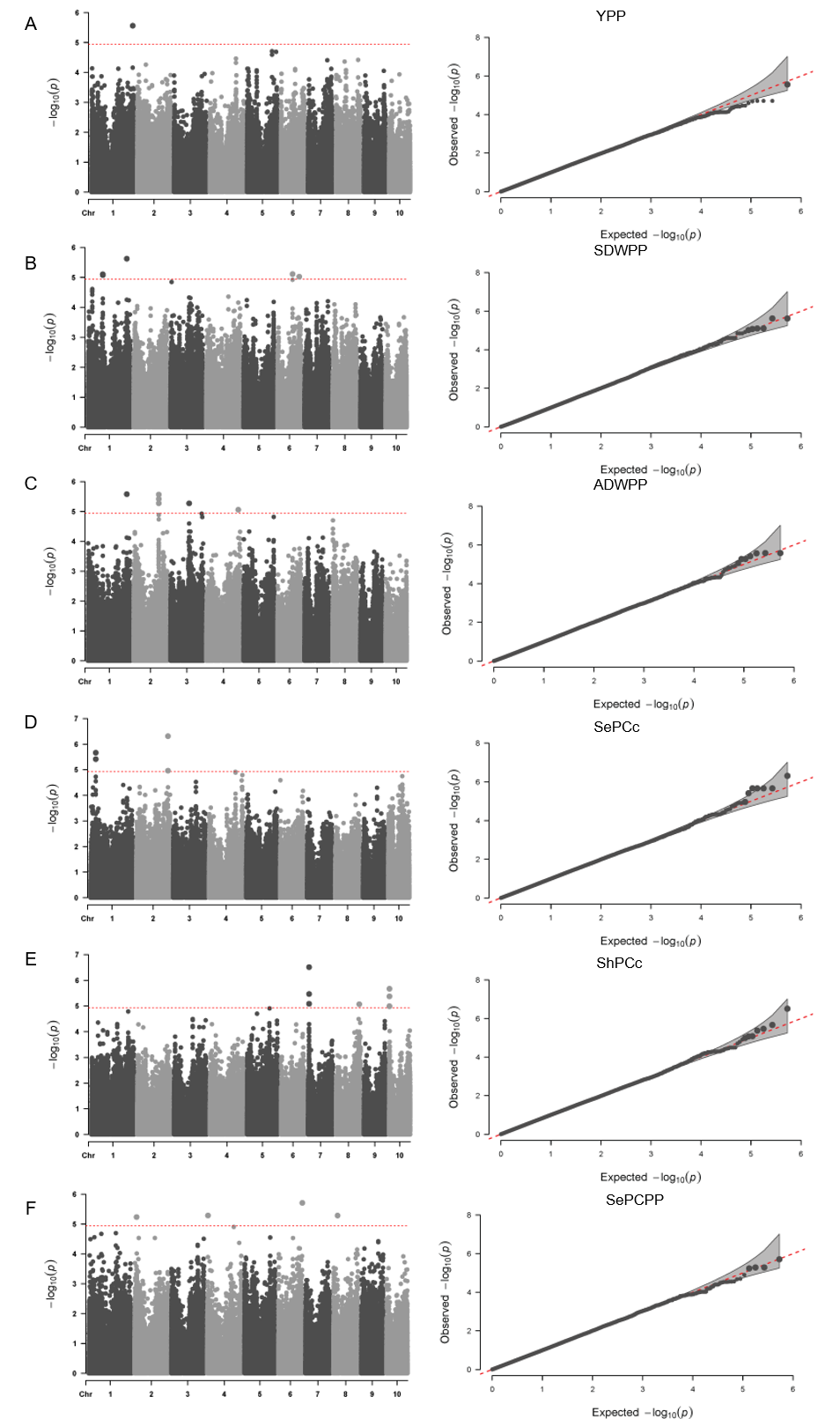

Supplement: Supplementary file 1 [file ijms-22-09311-s001.zip › Supplementary files_proofreading_20210827/Figure S6-Part1.png]

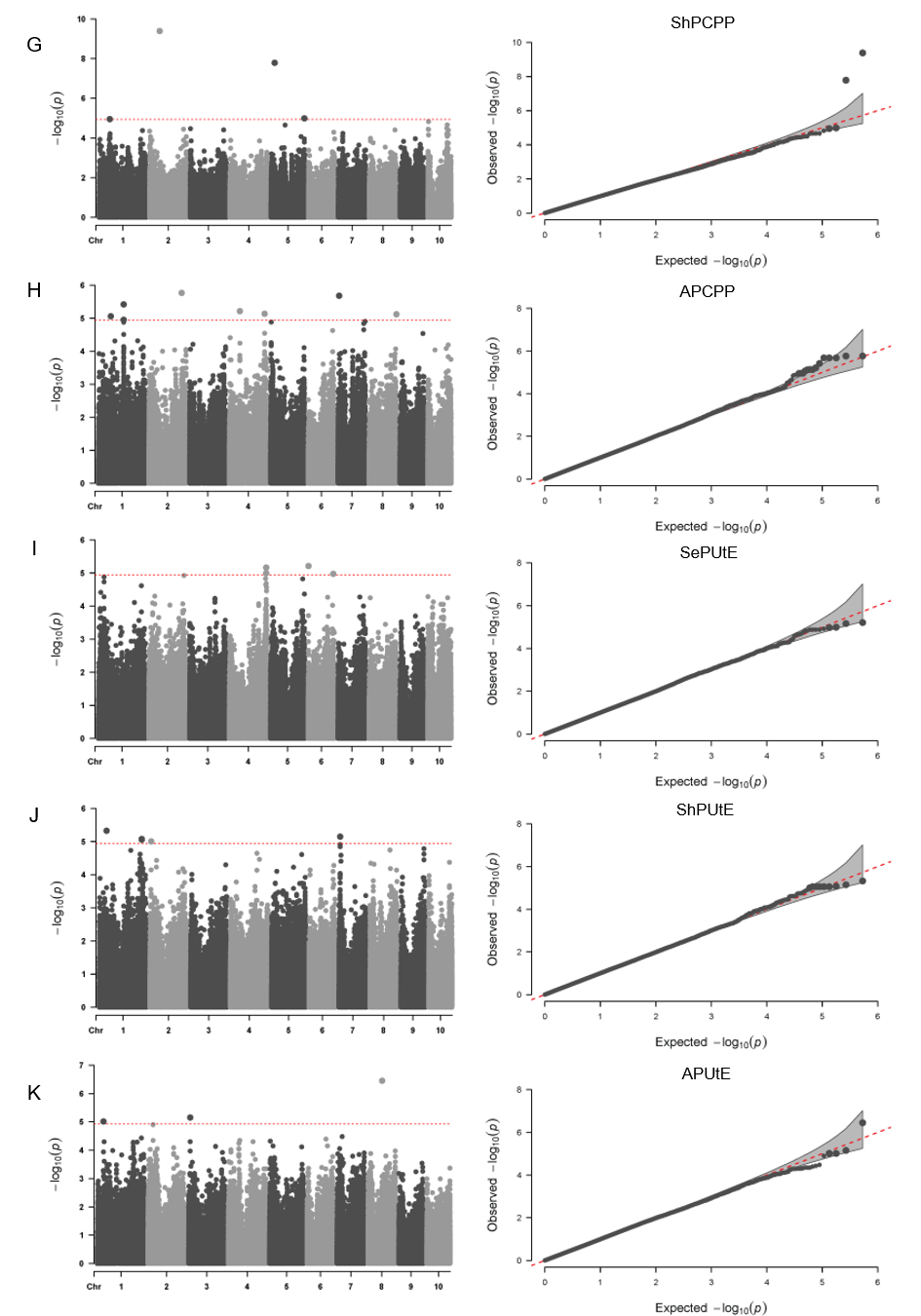

Supplement: Supplementary file 1 [file ijms-22-09311-s001.zip › Supplementary files_proofreading_20210827/Figure S6-Part2.png]

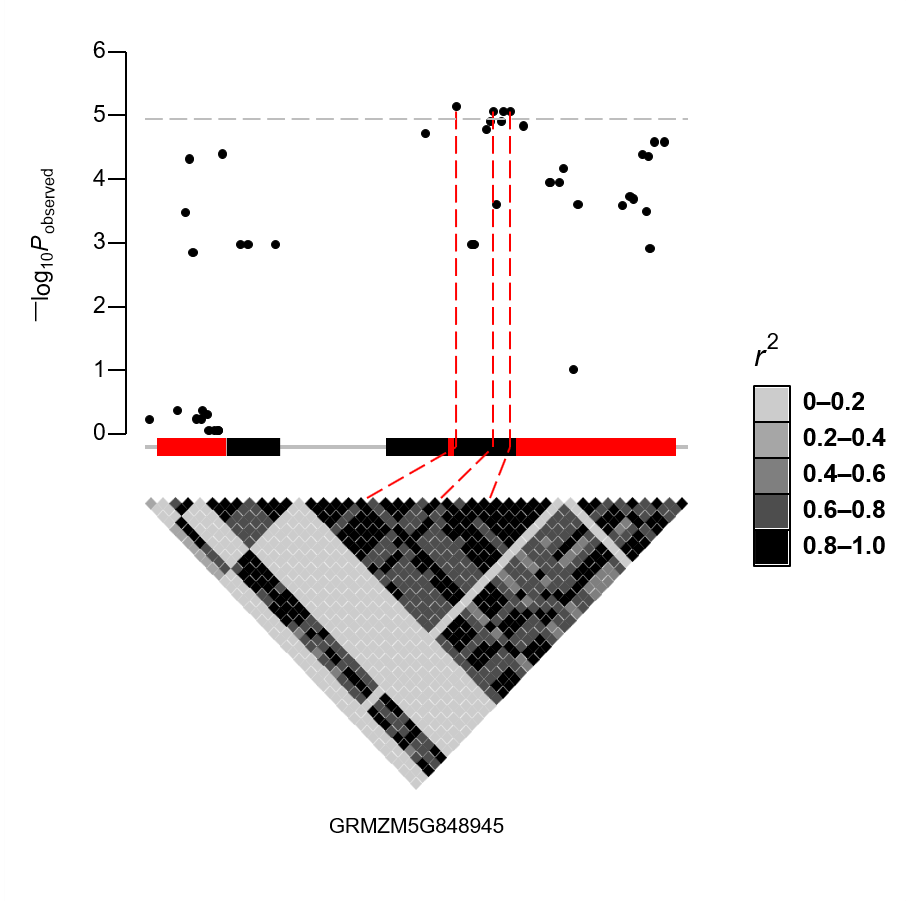

Supplement: Supplementary file 1 [file ijms-22-09311-s001.zip › Supplementary files_proofreading_20210827/Figure S7.png]

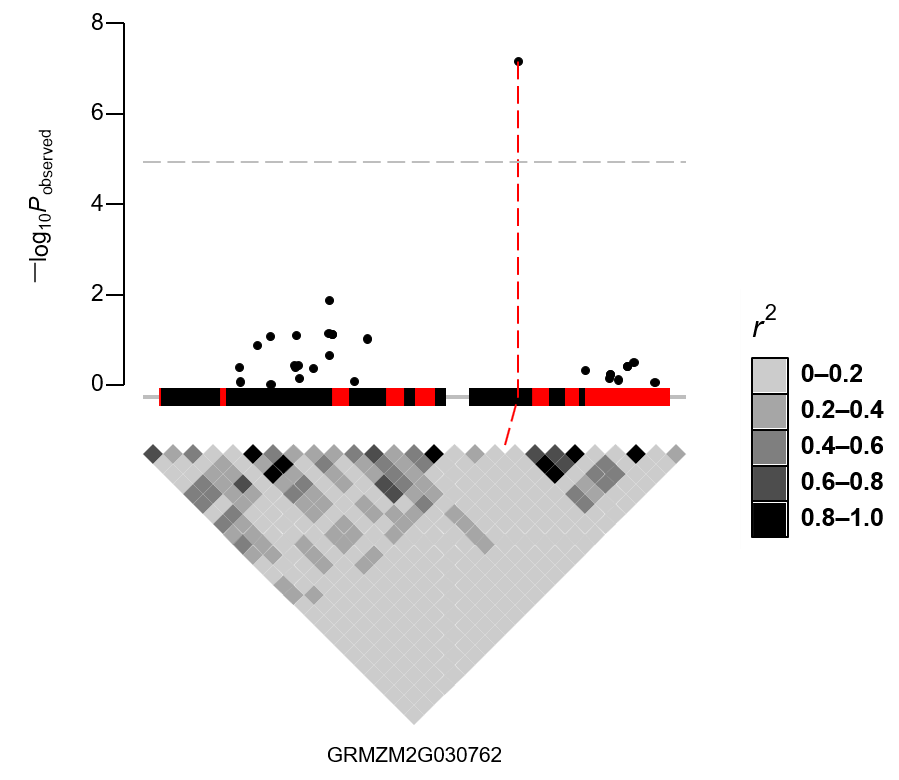

Supplement: Supplementary file 1 [file ijms-22-09311-s001.zip › Supplementary files_proofreading_20210827/Figure S8.png]

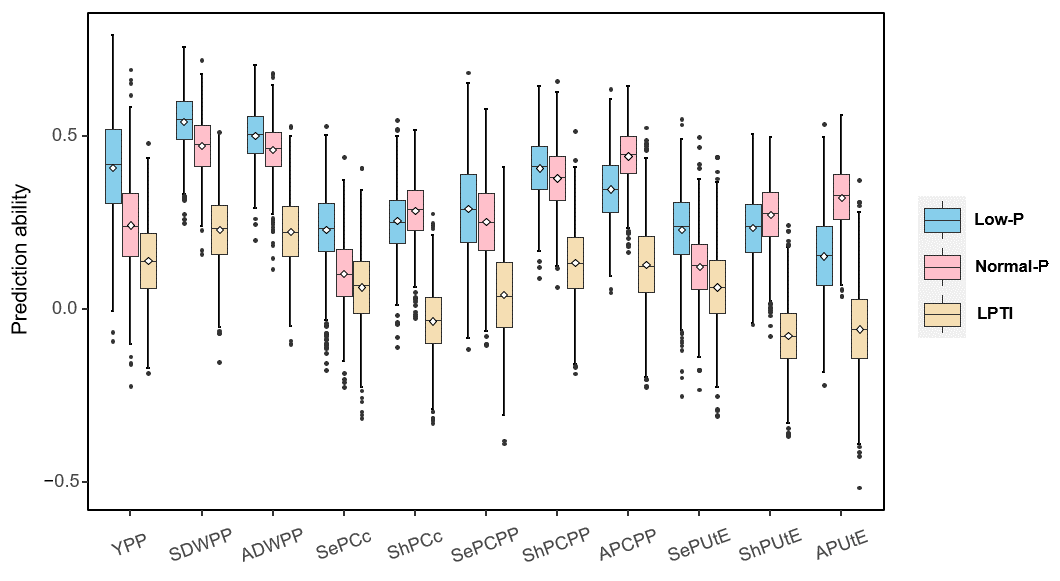

Supplement: Supplementary file 1 [file ijms-22-09311-s001.zip › Supplementary files_proofreading_20210827/Figure S9.png]
